# Are alexithymia and empathy predicting factors of the resilience of medical residents in France?

**DOI:** 10.5116/ijme.5ac6.44ba

**Published:** 2018-04-30

**Authors:** Audrey Morice-Ramat, Lionel Goronflot, Gilles Guihard

**Affiliations:** 1 Department of General Medicine, Faculty of Medicine, University of Nantes, France; 2 Center for Research in Education of Nantes (CREN), University of Nantes, France

**Keywords:** Burnout, coping, family medicine, medical formation, mental health, stress

## Abstract

**Objectives:**

To explore resilience, resilience predicting factors and resilience distribution in French medical residents.

**Methods:**

A cross-sectional study was conducted in which general practice residents (n = 380) were asked to answer the Jefferson Scale of Physician Empathy, the Connor-Davidson Resilience Scale, and the Toronto Alexithymia Scale. One hundred thirty-seven (137) responses were collected. The scores of the different scales have been calculated. The score differences were examined using the Student’s t-test or analysis of variance. The correlations were estimated using the Pearson correlation coefficient. The relationships between scores were analysed by multiple linear regression. The heterogeneity of the sample was examined by non-hierarchical cluster analysis.

**Results:**

Resilience and empathy were positively correlated (r_(135)_ = .36, p< .001). Alexithymia was negatively correlated with resilience, *r*_(135) _= -.40,p<.001, and empathy, *r*_(135) _= -.38, p<.001. Resilience was influenced by alexithymia, *b *= -.284, p = .001, empathy, *b*= .255, p = .002, gender (female < male), *b* = -.231, p = .002 and year of formation, *β*= .157, p = .036. Two clusters of residents were characterized. They differed by their empathy and resilience profiles and by alexithymia trait.

**Conclusions:**

Alexithymia, empathy, gender and year of formation correspond to predicting factors of resilience. This suggests that the resilience of vulnerable residents can be enhanced by increasing their empathy and by reducing their alexithymia. Thus, teaching teams could sustain their students’ well-being through educational programs aiming to develop their understanding of their own emotions and those of their patients.

## Introduction

Professional burnout (PBO) represents an inadequate response to chronic occupational stress.^1^ It results in psychological and physiological consequences that can strongly impact individual well-being, quality of life and job performance. Pathological signs of PBO include cardiovascular and gastrointestinal diseases.^2^^,^^3^ PBO is also accompanied by psychological symptoms such as anxiety, depression, motivational decrease, reduced interpersonal commitment and performance limitations.^4^ The PBO prevalence has been estimated in US working adults (~28%),^5^^,^^6^ in the general population of Sweden (~13%),^7^ and Germany (~4%).^8^ A growing body of evidence shows that PBO affects health care providers,^9^^-^^11^ with a prevalence depending upon factors including the geographic location and the specialty of the health profession.^12^^,^^13^ Burnout is also reported to impact health students during their training.^14^^-^^16^ The prevalence has been determined in different populations of medical students, showing that ~15% to ~45 % of students are affected.^16^^,^^17^ Medical residents are not spared from burnout. In particular, 25% of general practice residents are impacted by PBO in France.^18^

The resilience represents the ability to show positive psychophysiological outcomes despite experiencing aversive situations or living in a stressful environment.^19^ Improving the resilience of individuals has been evoked to prevent the PBO occurrence in a broad working context.^20^ Resilience and PBO are characterized by a negative correlation in health professions.^21^^-^^24^ Such a correlation is also reported for medical students.^25^^,^^26^ Numerous factors influence the resilience of individuals. These include age, gender, cultural environment, living perspectives and personality traits.^19^^,^^27^ Alexithymia corresponds to the impaired understanding of one’s own emotions.^28^ Alexithymia is observed in patients suffering from psycho-pathological diseases that reduce patient’s resilience.^29^^,^^30^ Negative correlations between alexithymia and resilience have been characterized in Chinese military personnels^31^ and in Iranian students.^32^ Empathy can also represent a factor influencing the resilience. Empathy represents the ability to experience other’s emotions and to manifest a cognitive adaptation to promote better interpersonal relationships.^33^ Indirect clues suggest a cross-talk between empathy and resilience.^34^ However, the existence of a significant correlation between empathy and resilience is still discussed.^35^^,^^36^

The distribution of resilience among populations was previously assessed by using cluster analysis procedure. Pietrzak and Cook reported the existence of three clusters of individuals with distinct resilience levels among a sample of US veterans.^37^ Doron and colleagues identified five clusters of students differing by their coping strategies in response to stress.^38^ The work of Suriá Martínez indicated the existence of different resilience profiles among a sample of patients with spinal cord injury.^39^ Taken together, these works suggest that the resilience is not distributed according to a single normal distribution. This is also the case for empathy and alexithymia distributions.^40^^,^^41^

Although resilience measurement has been described in medical students and in medical residents,^25^^,^^26^^,^^42^ little is known about the predicting factors of resilience. Furthermore, there is a lack of study concerning the characterization of resilience and resilience predicting factors in French health students. Therefore, the objective of the present study is to explore the resilience of medical students. To do so, a survey was conducted to measure resilience, to determine the nature of predicting factors, and to characterize the distribution of resilience among general practice residents at Nantes University (France). It was hypothesized that: i) the resilience is affected by predicting factors including empathy and alexithymia, and ii) the resilience distribution among a student sample is heterogeneous.

## Methods

### Study design and participants

A cross-sectional study was conducted at Medical School of Nantes University (France). Ethical approval was obtained from the Ethics committee of Nantes University. Project staff was not involved in the diploma allocation. All general practice residents (n=380) were eligible to the study. The return rate was ~40% (n=150). Thirteen incomplete answers or with outlying scores (Dixon’s test) were discarded. The final sample (n=137, mean age =26.5, SD=1.3) corresponded to 94 women (mean age =26.6, SD=1.2) and 43 men (mean age =26.5, SD =1.6). The proportions of residents registered in years 1, 2 and 3 of the formation corresponded to ~30% (n = 41), ~39% (n=53) and ~31% (n=43). The gender ratio (F/M) differed significantly between years 1 (25/16), 2 (43/10) and 3 (26/17) (χ^2^(2, N=137) = 6.293, p=.043). Becoming a general practice resident was a deliberate choice for 123 respondents (~90%).

### Study tools

The questionnaire included items related to socio-demographic information (gender, age, year of the curriculum). Items from the French versions of the Jefferson Scale of Physicians Empathy (f-JSPE; 25 items), the Connor-Davidson Resilience Scale (f-CDRISC; 21 items) and the 20-item Toronto Alexithymia Scale (f-TAS20) was also included in our survey. These scales have demonstrated satisfactory reliability for the assessment of resilience, empathy, and alexithymia.^43^^-^^45^

The scoring of f-JSPE items is based on a 7-point Likert scale with one corresponding to “full disagreement” and seven corresponding to “full agreement”.^10^ items needed reverse scoring because of their formulation. Total f-JSPE score varies from 20 to 140; a high score indicates high empathy. F-CDRISC and f-TAS20 scales use a 5-point Likert scale for item scoring. For f-CDRISC, the item score varies from 0 (“full disagreement”) to 4 (for “full agreement”), and the total score varies from 0 to 84. For f-TAS20, the item score varies from 1 (for “full disagreement”) to 5 (for “full agreement”). Five items needed reverse scoring. Total f-TAS20 score varies from 20 to 100. High scores for f-CDRISC and f-TAS20 indicate high resilience and alexithymia traits.

### Data collection procedures

All general practice residents were contacted by e-mail. They were invited to answer an electronic version of the questionnaire. The access to the questionnaire was granted after the validation of an electronic informed consent in which goals, means, and methods have been described. To ensure resident’s anonymity, each resident encoded an identifier by using the first two letters of his/her first name, a number corresponding to the day of birth (between 01 to 31), a number corresponding to the year of birth (between 00 and 99), a number corresponding to the place of birth (between 00 and 101) and the first two letters of his/her mother’s given name.

### Data analysis

Data were collected at the end of the survey. They were analysed with SPSS 21, Sigma Plot 12 and R (3.2.5) software. F-CDRISC scale has recently been validated for resilience measurement in health students.^43^ The reliability of f-JSPE and f-TAS20 scales was re-assessed in our conditions. The item-score correlation coefficient (rIS) was calculated. As Cronbach’s α coefficient is not a good estimator of internal consistency for multifactorial scales,^46^ greatest lower bound (GLB) and McDonald’s w coefficient were calculated as recommended elsewhere.^47^ The scale appropriateness was deduced from the Kaiser-Meyer-Olkin coefficient (KMO, optimal value above .8) and the Bartlett’s test (optimal p < .001). The item-sampling adequacy was deduced from the anti-image correlation coefficient value calculated for each item (AIC, optimal value above .5).

To confirm the 3-factor structure of the f-JSPE and f-TAS20 scales, a confirmatory factor analysis (CFA) was performed by using the maximum likelihood method. The goodness of fit of a factor model was estimated according to Byrne’s recommendations.^48^ The following indices were calculated: i) the normed χ^2^ (χ^2^ /df, optimal value below 2.0), ii) the standardized root mean square of residuals (sRMR, optimal value below .05), iii) the goodness of fit index (GFI) and the adjusted goodness of fit index (AGFI), both being optimally higher than .90), iv) the root mean square error of approximation (RMSEA, optimal value below .08) and its relative p(close) for which a value above .05 indicates a good fit, and v) the comparative fit index (CFI) (optimal value above .90).

The normality of data distribution was verified by using the Shapiro-Wilk’s test (significance criterion p>.05). Mean (M), standard deviation (SD), 95% confidence interval (95% CI) and the average score (i.e., the ratio between the total score and the number of items of a scale) were calculated for the different scales and different subgroups. A correlation between two variables was deduced from the value of the Pearson correlation coefficient. The differences were estimated by Student’s t-test (for two-group comparison) and by analysis of variance (ANOVA, for multiple-group comparison) in considering a risk α = .05. The threshold of significance of a difference was set at p<.05. When a difference was significant, the effect size was estimated by Cohen’s d coefficient (for two-group comparison) with the correction of Rosnow and Rosenthal^49^ or by η^2^ coefficient (for multiple-group comparison). Small, medium and large effect sizes were respectively characterized by d, η^2^ ≤ .2, .2 < d, η^2^ ≤ .5 and .5 < d, η^2^.^50^

Multiple linear regression analyses were performed to test whether alexithymia and empathy can predict resilience. Gender (male = 1, female = 2) and year of formation (year 1 = 1, year 2 = 2 and year 3 = 3) were also considered as potential predicting factors of resilience. Standardized regression coefficient (*b*) and p values were calculated to estimate the relationships between the different variables.

The heterogeneity within a dataset can be ascertained by cluster analysis (CA).51 A non-hierarchical CA (K-means) was run by using standardized scores (z scores) for f-JSPE, f-CDRISC and f-TAS20 as clustering variables. The validity of different models (from 2 to 4 clusters) was assessed. The significance of between-cluster differences was calculated by unpaired Student’s t-test (for a 2-cluster model) or by ANOVA and post-hoc Bonferroni correction (for 3- and 4-cluster models). The most likely cluster model contained the highest number of clusters for which all z scores were significantly different. The validity of the retained model was assessed by discriminant analysis (DA), in which z scores were considered as independent variables, whereas the number of clusters corresponds to the dependent variable. For cluster and discriminant analyses, the significance criterion was set at p < .001.

## Results

The normality of f-JSPE and f-TAS20 scores was confirmed using the Shapiro-Wilk test. Therefore, a factor analysis using maximum likelihood method and an orthogonal rotation (Varimax) could be performed to determine the indicators of reliability, as suggested by Costello and Osborne.^52^ As shown in Table 1, f-JSPE and f-TAS20 scales were characterized by acceptable to good appropriateness, item-sampling adequacy, and item-score correlation. The internal consistency was considered from acceptable (for f-JSPE) to strong (for f-TAS20) as demonstrated by GLB and wvalues. A 3-factor structure was determined for both f-JSPE and f-TAS20 scales, as illustrated by the values of goodness-of-fit indices (Table 1).

### Scores analysis

As shown in Table 2, f-CDRISC was significantly best-scored by male residents (low effect size). A significant difference in resilience (low effect size) was observed between the residents of the different years of formation. However, our analysis indicated that such a difference originated more likely from an interaction between gender and year of formation. Gender-related or curriculum-related differences for f-JSPE or f-TAS20 scores were not significant.

### Multiple linear regression analysis

Resilience and empathy were positively correlated, r_(__135)_ = .36, p<.001. Negative correlations were observed between alexithymia and resilience, r_(__135)_ = -.40, p<.001, and between alexithymia and empathy, r_(135)_ = -.38, p<.001. A regression model describing the contribution of the different variables to the resilience was elaborated. It was supported by a significant regression equation, R^2^_adjusted_ = .27, F_(__4,132)_ = 13.39, p< .001, Durbin-Watson coefficient = 1.625. The resilience was positively predicted by empathy, *b* = .255, t_(__132)_ = 3.19, p=.002, and by year of formation, *b* = .157, t_(132)_ = 2.12, p=.036. Meanwhile, gender (female < male, *b *= -.231, t_(__132)_ = -3.14, p= .002) and alexithymia, *b *=-.284, t_(132)_=-3.57, p < .001, corresponded to negative predicting factors. Alexithymia negatively influenced empathy, *b *=-.270, t_(__132)_ = -3.21, p=.002. Neither alexithymia nor empathy was affected by gender or by year of formation. This model was validated by CFA as demonstrated by the goodness-of-fit indices, χ^2^(5, N = 137)/df = .55, GFI = .99, AGFI = .98, sRMR = .042, RMSEA < .001, p(close) = .846.

### Cluster analysis

Different convergent solutions resulting from CA were observed. However, ANOVA shows that the differences produced by 3-and 4-cluster models were not significant (data not shown). For this reason, the 2-cluster model was considered as the most reliable solution. This was validated by DA. The cluster effective calculated by DA were found to be identical to those determined by CA. A very strong correlation between the allocations determined by CA and by DA was observed, r(135) =.98, p< .001.

**Table 1:** Determination of the psychometric properties of empathy and alexithymia scales

Scale	KMO	Bartlett’s test	AIC range	Mean rIS (135) (SD) [95% CI]	Confirmatory analysis						Indicators of internal consistency	
					^2^/df	sRMR	GFI	AGFI	RMSEA	CFI	GLB	ω [95% CI]
f-JSPE	0.79	χ^2^(190, N = 137) = 598.8, p < 0.001	0.608 - 0.872	0.29 (0.15) [0.22-0.36]	1.32	0.07	0.87	0.84	0.047	0.88	0.87	0.70 [0.67-0.74]
f-TAS20	0.814	χ^2^(190, N = 137) = 830.9, p < 0.001	0.610 - 0.911	0.42 (0.16) [0.34-0.50]	1.34	0.07	0.87	0.83	0.05	0.92	0.91	0.84 [0.81- 0.88]

AGFI: Adjusted Goodness-of-Fit Index; AIC: anti-image coefficient; CFI: Confirmatory Fit Index; χ^2^/df: normed χ^2^; df: degree of freedom; GFI: Goodness-of-Fit Index; GLB: Greatest Lower Bound coefficient; rIS: item-score correlation coefficient; KMO: Kaiser-Meyer-Olkin coefficient; ω: McDonald’s ω coefficient; RMSEA: Root Mean Square Error of Approximation; sRMR: standardized Root Mean Square of Residuals; [95% CI]: 95% confidence interval.

The gender repartition determined for each cluster was similar (Table 3). By contrast, between-cluster differences calculated for resilience, empathy, and alexithymia were significant. The residents of cluster 1 were more resilient and more empathetic than those of cluster 2 (strong effect size). The residents allocated to cluster 2 exhibited a higher alexithymia profile (strong effect size). The between-cluster difference was also observed when the year of formation was considered as comparison criterion. In particular, residents from 3rd year were more abundant in cluster 1, whereas cluster 2 was mainly constituted by 1st and 2nd-year residents.

## Discussion

The objective of the present study consisted in a better understanding of the characteristics of resilience of medical students. Our work was devoted to the measure of resilience, to the characterisation of some resilience predicting factors and to the analysis of the resilience distribution in a sample of French general practice residents. Two working hypotheses have been tested: i) the resilience is affected by several predicting factors including empathy and alexithymia and ii) the distribution of resilience among the studied sample is heterogeneous.

### Characterization of the resilience

To explore our first working hypothesis, f-JSPE and f-TAS20 scales are used for empathy and alexithymia measurements. These scales have been described as reliable tools in different French-speaking samples.^44^^,^^45^^,^^53^^-^^55^ However, a psychometric scale needs *de novo* validation when study conditions are changed.^52^ Our work confirms that f-JSPE and f-TAS20 correspond to reliable tools for empathy and alexithymia measurements in French medical residents.

The average score of alexithymia calculated for French medical residents is similar to those calculated for French asymptomatic adults,^45^ and for German and Japan general populations.^56^^,^^57^ There are contradictory results concerning a gender-related difference for alexithymia in the literature. Indeed, previous observations highlight some gender-related differences in German and Jordanian general populations.^56^^,^^58^ However, other reports show that alexithymia is unaffected by the gender of French or Japan individuals.^45^^,^^59^^,^^60^ Our study shows that the gender of French medical residents does not influence their alexithymia trait.

The average score of empathy calculated in the present work is in good agreement with those calculated for medical students^61^ and for French medical practitioners.^54^^,^^62^ The fact that empathy level is affected by gender or not, is currently debated. On the one hand, a higher empathy is reported for females in samples of undergraduate students of Portugal and USA.^63^^,^^64^ On the other hand, North American and Brazilian male residents have higher empathy levels than their female equivalents.^65^^,^^66^ The present study does not report any gender-related empathy difference for French medical residents. This suggests that medical formation at Nantes University minimizes or abolishes the empathy difference between male and female medical students during their early training.

The resilience measured for French medical residents is higher than that determined for Chinese, Korean and US general populations.^67^^-^^69^ By contrast, it is close to that measured for Australian nurses or Brazilian athletes daily living in stressful conditions^70^^,^^71^ and for Chinese and Turkish eartquake survivors.^72^^,^^73^ This indicates that medical formation provides a training environment susceptible to support the resilience of medical students.

The relationships between resilience, empathy, and alexithymia have never been fully described, because of a lack of concomitant measurements. It is shown in the present work that empathy and alexithymia correspond to significant predicting factors of resilience. Indeed, resilience is negatively influenced by alexithymia and positively affected by empathy which is also negatively influenced by alexithymia. A decline of medical students’ empathy occurs as students progress in their training.^74^ Our findings suggest that such a decline can be accompanied by the decrease of the students’ resilience, thus rendering the students more vulnerable to PBO. Apart from training programs suggested for resilience enhancement,^75^^,^^76^ education programs devoted to the reinforcement of students’ empathy could represent an efficient strategy aiming to support resilience. Also, helping students in the understanding of their own emotions could also figure a protection factor against PBO.

**Table 2:** Resilience, empathy and alexithymia of general practice residents

Variable	f-CDRISC	f-JSPE	f-TAS20
Overall (n=137)	3.17 (.73) [2.77 – 3.57]	5.61 (.43) [5.44 – 5.73]	2.38 (.54) [2.23 – 2.58]
Female (n=94)	2.79 (.70) [2.57 – 3.01]	5.63 (.41) [5.44 – 5.72]	2.38 (.54) [2.28 – 2.62]
Male (n=43)	3.34 (.63) [3.17 – 3.57]	5.57 (.47) [5.44 – 5.73]	2.37 (.56) [2.17 – 2.53]
Gender Comparison (d)	t_(__135)_ = 2.09 , p = .038 (.12)	t_(__135)_ = .69 , p = .408	t_(__135)_ = .01 , p = .918
Year-1 (41)	3.13 (.67) [2.92 – 3.34]	5.57 (.49) [5.46 – 5.71]	2.40 (.56) [2.23 – 2.58]
Year-2 (53)	2.79 (.69) [2.57 – 3.00]	5.57 (.47) [5.41 – 5.72]	2.41 (.54) [2.24 – 2.57]
Year-3 (43)	3.49 (.63) [3.29 – 3.69]	5.69 (.40) [5.56 – 5.82]	2.27 (.55) [2.09 – 2.44]
Between-year Comparison (η^2^)	F_(__2, 134)_ = 6.10, p < .001 (.18)	F_(__2, 134)_ = .60, p = .549	F_(__2, 134)_ = .47, p = .628
Gender x Year Comparison	F_(__1, 131)_ = 3.70, p = .027	F_(__1,131)_ = 2.25, p = .109	F_(__1, 131)_ = 2.12, p = .124

Data correspond to average scores (SD) and 95% confidence interval ([95% CI]) calculated for the overall sample and different sub-samples. d and η2: effect size of differences.

**Table 3:** Characteristics of the clusters resulting from cluster and discriminant analyses

Analysis	Characteristics	Cluster 1	Cluster 2	test, significance (d)
	N	54	83	
Cluster analysis	f-CDRISC	3.58 (.62)	2.81 (.64)	t_(__135)_= -6.99, p< .001 (1.22)
	f-JSPE	5.91 (.31)	5.42 (.38)	t_(__135)_= -8.06, p< .001 (1.44)
	f-TAS20	1.88 (.35)	2.70 (.38)	t_(__135) _= 12.91, p< .001 (2.27)
Discriminant analysis	N	54	83	
	f-CDRISC	3.58 (.62)	2.81 (.63)	t_(__135)_= -6.95, p< .001 (1.23)
	f-JSPE	5.91 (.31)	5.43 (.38)	t_(__135)_= -7.82, p< .001 (1.40)
	f-TAS20	1.87 (.34)	2.70 (.38)	t_(__135)_= 13.15, p< .001 (2.33)
Gender (F - M)		36 - 18	58 - 25	χ^2^(1, N=137) =.04, p=.84
Year of formation (%)	1	14 (25.9)	27 (32.5)	χ^2^(2, N=137) =7.21, p=.03
	2	16 (29.6)	37 (44.6)	
	3	24 (44.5)	19 (22.9)	

The data correspond to average scores (SD). P value indicates the significance. The effect size is given by Cohen’s coefficient (d).

### Analysis of the distribution of resilience

Cluster analysis has been used to describe heterogeneous distributions of empathy,^64^ alexithymia^57^^,^^77^ and resilience^37^ among pathological and non-pathological populations. However, these results have been obtained with independent measurements of empathy, alexithymia or resilience. In the present study, empathy, resilience, and alexithymia are concomitantly measured, and cluster analysis considers the three variables simultaneously. Two clusters of residents are identified in very good conditions of confidence. The former is mainly composed of 3rd-year residents with low alexithymia trait and high empathy and resilience. The latter is predominantly constituted by 1st and 2nd-year residents with a high alexithymia propensity and low empathy and resilience. This suggests that the medical residency training increases the emotional understanding and the resilience of the residents.

Considering normative data,^61^^,^^69^ the residents of both clusters exhibit non-pathological levels of empathy and resilience. However, a TAS20 score higher than 2.65 is reported to indicate a strong alexithymia propensity.^56^ Consequently, our work suggests that the residents of the second cluster have a high alexithymic profile. Therefore, this work corroborates Shapiro’s comments concerning the promotion of students’ alexithymia during preclinical and clinical medical formation.^78^ Predictive determinants of alexithymia during early stages of medical training shall be investigated in future studies.

### Limitations

Although significant datasets support our work, it presents several limitations. It corresponds to a monocentric study (Faculty of Medicine of Nantes). The results are based on a limited number of responses (49% of the population of residents). A selection bias may be present in the survey, as collected responses can originate from students with a positive a priori for this study. Furthermore, medical residents are potentially able to identify socially acceptable answers concerning the level of empathy associated with medical practice. Fisher and Katz describe this social desirability-bias on self-report assessment.^79^ It may be responsible for some overestimated responses and the present survey. The last limitation concerns the design of the study which corresponds to a cross-sectional survey. This prevents the observation of time-related changes in empathy, resilience, and alexithymia. Consequently, further longitudinal investigations based on larger samples obtained from different medical schools are necessary.

## Conclusions

Our study corresponds to the first concomitant analysis of resilience, empathy and alexithymia traits in a sample of French general practice residents. It is shown that empathy, alexithymia, year of formation and gender are predicting factors of resilience. Our work suggests that it is possible to sustain the resilience of residents by acting on their empathy and alexithymia. Our study shows that residents can be segmented into two subgroups of distinct profiles concerning their empathy and resilience and their alexithymia. Medical training teams should consider these findings to improve their teaching strategies.

### Conflict of Interest

The authors declare that they have no conflict of interest.
